# The Multifactorial Pathogenesis of Endometriosis: A Narrative Review Integrating Hormonal, Immune, and Microbiome Aspects

**DOI:** 10.3390/medicina61050811

**Published:** 2025-04-27

**Authors:** Zaure Datkhayeva, Ainur Iskakova, Alla Mireeva, Aida Seitaliyeva, Raikhan Skakova, Gulshat Kulniyazova, Aiman Shayakhmetova, Gaukhar Koshkimbayeva, Chapen Sarmuldayeva, Lazzat Nurseitova, Lyailya Koshenova, Gulzhan Imanbekova, Dina Maxutova, Sandugash Yerkenova, Aigerim Shukirbayeva, Ulzhan Pernebekova, Zaure Dushimova, Akerke Amirkhanova

**Affiliations:** 1 Department of General Medical Practice No. 2, School of Medicine, S.D. Asfendiyarov Kazakh National Medical University, Tole-bi 94, Almaty 050012, Kazakhstan; ferizat2008@mai.ru (Z.D.); mireeva.a@kaznmu.kz (A.M.); 70.lazzat@mail.ru (L.N.); dina.zhusup@mail.ru (D.M.); sanduka-85@mail.ru (S.Y.); 2 Department of Public Health and Social Sciences, Kazakhstan’s Medical University “KSPH”, Utepov str.19A., Almaty 050000, Kazakhstan; 3 Higher School of Medicine, Al-Farabi Kazakh National University, Tole-bi 96, Almaty 050040, Kazakhstan; seitaliyeva.aida@med-kaznu.com (A.S.); kulniyazova.gulshat@med-kaznu.com (G.K.); shayakhmetova.aiman@med-kaznu.com (A.S.); dushimova.zaure@med-kaznu.com (Z.D.); 4 Department of Obstetrics and Gynecology, School of Medicine, S.D. Asfendiyarov Kazakh National Medical University, Tole-bi 94, Almaty 050012, Kazakhstan; raikhan12.07@mail.ru (R.S.); lyalya_69@mail.ru (L.K.); aikerim.almuratova19@mail.ru (A.S.); ulzhan08.92@mail.ru (U.P.); 5 Department of General Medical Practice with Courses, Kazakh-Russian Medical University, Abylai Khan 51/53, Almaty 050000, Kazakhstan; gauhar.24@mail.ru; 6 City Center for Human Reproduction, st. Zhibek-Zholy 7W6M+CQR, Almaty 050000, Kazakhstan; chapen@mail.ru; 7 “Almaty City Hospital No. 29” Communal State Enterprise on the Right of Economic Management, Microdistrict Zerdeli 371/3, Almaty 050000, Kazakhstan; gulzhanimanbekova@gmail.com; 8 School of Pharmacy, S.D. Asfendiyarov Kazakh National Medical University, Tole-bi 94, Almaty 050012, Kazakhstan

**Keywords:** endometriosis, gut microbiota, estrogen, inflammation, microbial, probiotics, fecal microbiota transplantation

## Abstract

Endometriosis (EM) is a common estrogen-dependent chronic inflammatory disorder affecting reproductive-aged women, yet its pathogenesis remains incompletely understood. Recent evidence suggests that the gut microbiota significantly influence immune responses, estrogen metabolism, and systemic inflammation, potentially contributing to EM progression. This narrative review explores the relationship between the gut microbiota and EM, emphasizing microbial dysbiosis, inflammation, estrogen regulation, and potential microbiome-targeted therapies. Studies published within the last 30 years were included, focusing on the microbiota composition, immune modulation, estrogen metabolism, and therapeutic interventions in EM. The selection criteria prioritized peer-reviewed articles, clinical trials, meta-analyses, and narrative reviews investigating the gut microbiota’s role in EM pathophysiology and treatment. Microbial dysbiosis in EM is characterized by a reduced abundance of beneficial bacteria (*Lactobacillus, Bifidobacterium,* and Ruminococcaceae) and an increased prevalence of pro-inflammatory taxa (*Escherichia/Shigella, Streptococcus,* and *Bacteroides*). The gut microbiota modulate estrogen metabolism via the estrobolome, contributing to increased systemic estrogen levels and lesion proliferation. Additionally, lipopolysaccharides (LPS) from Gram-negative bacteria activate the TLR4/NF-κB signaling pathway, exacerbating inflammation and EM symptoms. The interaction between the gut microbiota, immune dysregulation, and estrogen metabolism suggests a critical role in EM pathogenesis. While microbiota-targeted interventions offer potential therapeutic benefits, further large-scale, multi-center studies are needed to validate microbial biomarkers and optimize microbiome-based therapies for EM. Integrating microbiome research with precision medicine may enhance the diagnostic accuracy and improve the EM treatment efficacy.

## 1. Introduction

Endometriosis (EM) is a common estrogen-dependent chronic inflammatory gynecological disorder, affecting approximately 5 to 15% of women worldwide [1]. This condition is characterized by the ectopic growth of endometrial glands and stroma outside the uterus, which undergoes cyclical bleeding, proliferation, and fibrosis in response to estrogen stimulation [2,3,4]. These pathological changes contribute to a persistent inflammatory environment that significantly impacts women’s health [5]. The progression of EM is closely linked to the recruitment of proinflammatory cytokines and alterations in circulating immune cell populations, leading to chronic pelvic inflammation and affecting the surrounding structures [6]. Modern medicine describes the symptoms of EM as highly variable, ranging from severe dysmenorrhea, chronic pelvic pain, and heavy menstruation to gastrointestinal and urinary disturbances, fatigue, and depression [7]. This wide spectrum of symptoms, combined with the high heterogeneity and complexity of EM pathogenesis, often leads to delayed diagnosis and challenges in effective management. Consequently, the lack of specific symptoms, along with the absence of reliable diagnostic markers, deteriorates early detection, delays accurate diagnosis, and complicates effective treatment strategies, often resulting in prolonged suffering and reduced quality of life for affected individuals [5,8].

According to various epidemiological studies, the prevalence and impact of EM continue to escalate, highlighting the increasing burden of the disease on women’s health and healthcare systems worldwide. For example, the World Health Organization (WHO) defines EM as a multifaceted condition that impacts women of reproductive age, with an estimated global prevalence of 10% [9]. Nevertheless, the actual prevalence of EM may be underestimated, as empirical studies suggest that approximately 10–15% of women of reproductive age and between 35 and 50% of individuals experiencing pelvic pain or infertility are impacted by this condition [10]. Moreover, an estimated 30–50% of women diagnosed with EM may experience challenges related to infertility, as the pathology can result in pelvic adhesions, tubal scarring, inflammatory responses, hormonal dysregulation, immune system anomalies, and compromised oocyte quality, frequently leading to delays in conception or instances of unexplained infertility, ultimately exacerbating psychological distress [11].

While EM predominantly affects women from menarche to menopause, with a notable peak incidence occurring between the ages of 25 and 45, it has also been recognized in both adolescent populations and postmenopausal women [12]. Surgical investigations indicate that 0.1–53% of women who undergo a laparoscopy or laparotomy are found to have EM, with diagnoses being made in 12–32% during laparoscopic assessments for pelvic pain and in 10–60% of those undergoing laparoscopy for infertility-related concerns [12,13,14,15]. Additionally, genetic factors contribute to approximately 7% of cases, with EM identified in 2% of women who have undergone tubal ligation and in 17% of those who have had ovarian surgical procedures [16,17].

The exact etiology of EM remains undetermined, despite numerous theories attempting to explain its pathogenesis. The most widely accepted hypothesis, Sampson’s theory of retrograde menstruation, suggests that endometrial tissue fragments travel through the fallopian tubes into the peritoneal cavity, where they implant and proliferate [18]. However, this theory fails to account for the presence of deep-infiltrating EM or endometriotic lesions in extra-pelvic organs, including the lungs, brain, and gastrointestinal tract. Furthermore, retrograde menstruation occurs in nearly 90% of menstruating women, yet only a subset develops EM, suggesting that additional factors must contribute to disease onset and progression [19].

Alternative theories attempt to address these limitations. Gruenwald’s celomic metaplasia theory proposes that the peritoneal epithelium transforms into endometrial-like tissue under the influence of inflammatory cytokines and growth factors [20]. This hypothesis provides a plausible explanation for cases in women who lack a uterus, such as those with Mayer–Rokitansky–Küster–Hauser syndrome or postmenopausal patients [21]. However, it does not fully explain hormonal influences, genetic predisposition, or the invasive characteristics of endometriotic lesions, which resemble malignancies in their ability to establish a microvascular network and evade immune surveillance [22]. Similarly, the embryogenetic theory posits that ectopic endometrial tissue originates from embryonic remnants under the influence of estrogen, yet this fails to explain the heterogeneous presentation and progression of the disease [23].

The stem cell theory suggests that endometrial and hematopoietic stem cells migrate and differentiate into endometrial-like lesions, contributing to the ectopic implantation of endometrial tissue [24]. While this hypothesis aligns with recent findings on circulating mesenchymal stem cells, it does not elucidate why EM predominantly occurs in the pelvis rather than systematically, nor does it explain the high variability in disease severity among affected individuals [25]. Moreover, the emerging microbiome theory proposes that alterations in the gut and reproductive tract microbiota create a pro-inflammatory environment conducive to disease progression [26]. Although some studies suggest a potential role of bacterial endotoxins, the causative relationship remains unclear, and microbiota variations could be a consequence rather than a trigger of EM [27].

EM also exhibits hallmarks of immune dysregulation and genetic predisposition, prompting researchers to consider it an autoimmune-like disease [28]. Studies have identified increased somatic mutations, aberrant T-cell and B-cell activation, and chronic inflammatory responses in affected tissues [29]. However, unlike classical autoimmune disorders, EM lacks systemic autoantibodies and is largely confined to the peritoneal and reproductive organs [9]. Furthermore, exposure to endocrine-disrupting chemicals (EDCs), such as heavy metals and environmental pollutants, has been implicated in disrupting estrogen metabolism and immune function, possibly exacerbating EM [29]. Nevertheless, the precise role of EDCs remains controversial, with inconsistent findings in human studies [30].

Given these complexities, it is evident that no single theory can fully explain the pathogenesis of EM. The disease likely results from a multifactorial interplay of hormonal, genetic, inflammatory, immune, and environmental factors, making it a highly heterogeneous condition. The overlapping mechanisms among the proposed theories highlight the need for an integrated model that considers genetic susceptibility, immune dysfunction, hormonal imbalance, and epigenetic influences in disease development and progression [31].

Recent studies suggest that EM and the microbiota have a complex inter-relationship, with increasing evidence highlighting the importance of microbial dysbiosis in the progression of the disease [7,32]. Traditionally, the uterus was viewed as a sterile space; nonetheless, this idea has been thoroughly reassessed. New findings indicate that the uterus contains specific microbiota, questioning the traditional belief of a “sterile womb” [33]. The backward movement of menstrual fluid may additionally introduce microorganisms into the peritoneal cavity, worsening inflammation and aiding the development of ectopic lesions. Changes in the microbiota composition, known as dysbiosis, have been linked to various health issues, such as polycystic ovary syndrome (PCOS), infertility, inflammatory bowel disease (IBD), and tumors [34]. In the realm of EM, studies on non-human primates and mouse models have shown changes in the microbiota of the gut and reproductive system, marked by reduced *Lactobacilli* and a rise in Gram-negative bacteria, leading to heightened inflammation and immune system imbalance [35]. In addition, microbiota-depleted (MD) mouse models exhibited a reduction in the development of ectopic endometriotic lesions, an effect that was restored after fecal transplantation from mice suffering from EM, highlighting the possible influence of the gut microbiota on the progression of EM. The intestinal microbiome is essential for regulating estrogen; a disruption in this system can result in atypical estrogen metabolism, prolonged inflammation, and immune impairment, all crucial factors in the development of EM [36].

Since the microbiota composition influences various inflammatory and metabolic disorders, including EM, examining its role in reproductive health is crucial. Additionally, further research is needed to better understand the microbial environment in EM patients. In this review, we explore the intricate connection between the microbiota and EM, emphasizing a microbial imbalance as a potential factor in disease progression. Furthermore, we discuss microbiome-based strategies for disease prevention, early diagnosis, and therapeutic interventions, advocating for a comprehensive understanding of the microbiota’s role in EM progression and management.

## 2. Materials and Methods

**Figure 1:**
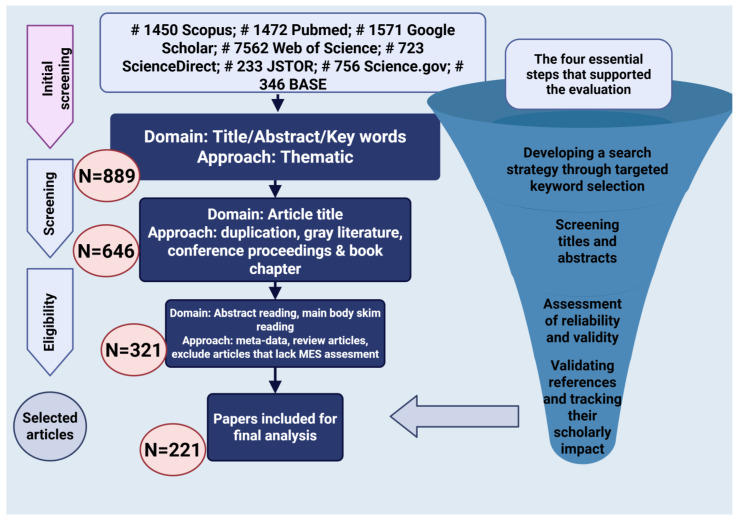
Flowchart of the article selection process and evaluation criteria for the narrative review.

This narrative review was performed in accordance with the PRISMA 2020 guidelines to guarantee transparency and reproducibility in the selection and screening processes [37]. The aim of this review was to explore the multifactorial origins of EM, with special emphasis on hormonal, immunological, and microbiota-associated factors. A thorough and methodologically sound approach was created to identify the pertinent literature, encompassing database selection, keyword development, phase organization, and the use of clearly outlined inclusion and exclusion criteria (Figure 1). The selection procedure was carried out following the four PRISMA stages: identification, screening, eligibility, and inclusion. At first, 12,320 records were found in eight databases, and following the elimination of duplicates and non-relevant entries, 889 articles were left for review. Following evaluations of the title, abstract, and full text, a total of 221 studies were incorporated into the final synthesis based on their scientific quality and relevance to the hormonal, immune, and microbial factors in the pathogenesis of EM.

### 2.1. Data Sources and Search Strategy

In January 2025, a comprehensive literature search was conducted across eight prominent academic databases: Public/Publisher MEDLINE (PubMed), Google Scholar, Scopus, Web of Science (WoS), ScienceDirect, Journal Storage (JSTOR), Science.gov, and Bielefeld Academic Search Engine (BASE). These databases were chosen to guarantee extensive coverage of peer-reviewed biomedical, clinical, and interdisciplinary studies. PubMed and WoS were favored for their reliable coverage of biomedical research and clinical studies, while Scopus and ScienceDirect offered extensive access to journals in the life sciences and medicine. Google Scholar was utilized to gather the gray literature and pertinent sources that are not indexed. JSTOR and BASE provided a broad multidisciplinary perspective, especially for historical and contextual analyses, while Science.gov granted access to medical research funded by the U.S. government. The exploration was directed by a thematic and organized keyword approach. The subsequent keywords and Boolean operators were utilized throughout all databases: “endometriosis”, “microbiota”, “microbiome”, “immune response”, “immune system”, “hormonal regulation”, “estrogen”, “gut bacteria”, “dysbiosis”, “pathogenesis”, “probiotic therapy”, “diagnosis”, and “treatment”.

Search queries were modified according to the specific syntax of each database as necessary. The results were handled with reference management software to guarantee the precise monitoring of duplicates and their inclusion status.

### 2.2. Eligibility Criteria

The explicit inclusion and exclusion criteria were utilized to maintain the relevance and scientific integrity of the literature featured in this review. The research was considered for inclusion if it was published in English, appeared in peer-reviewed journals from 1995 to 2025, and addressed endometriosis relating to microbiota, immune system processes, estrogen metabolism, or therapeutic strategies based on the microbiome. Permitted study designs comprised clinical trials, cohort studies, narrative reviews, and meta-analyses. Conversely, studies were omitted if they were in languages besides English, were duplicates found in multiple databases, or had publication dates earlier than 1995. Editorials, letters, commentaries, and articles that were not peer-reviewed were also omitted from the analysis. Furthermore, research that was entirely theoretical or did not have clinical significance was excluded. Papers that did not clearly discuss the link between microbiota and endometriosis, or that did not examine any of the key thematic areas such as immune factors, hormonal influences, or microbial interactions, were omitted from the final selection. This narrative strategy contributed to guaranteeing that the evaluation relied on quality and thematically relevant scientific data.

### 2.3. Quality Assurance

Each article was reviewed independently by two researchers, with any disagreements regarding inclusion settled through discussion or by consulting a third reviewer. Reference cross-checking and citation tracking were conducted to uncover any pertinent articles that may have been overlooked in the initial search stage. This repeated process guaranteed that the ultimate collection of evidence was thorough and scientifically sound.

## 3. Gut Microbiota Alterations in EM

The gut microbiota are integral to the preservation of holistic health and is subject to a multitude of host determinants, comprising dietary habits, lifestyle choices, chronological age, and environmental contexts, with dietary intake serving as a predominant modulator [38,39]. It facilitates vital physiological functions such as the regulation of appetite, extraction of energy, and metabolism of xenobiotics, thereby modifying the chemical configurations of dietary constituents, pharmaceuticals, and environmental toxins [40]. Recent studies underscore the robust correlation between the gut microbiota and immune system regulation, metabolic homeostasis, as well as the pathogenesis of a range of disorders, including obesity, non-alcoholic fatty liver disease (NAFLD), inflammatory bowel disease (IBD), cardiovascular disease (CVD), alcoholic liver disease (ALD), chronic kidney disease (CKD), and hepatocellular carcinoma [41]. Perturbations within the gut microbiota, referred to as dysbiosis, can adversely affect the gut–organ axis, thereby exacerbating disease development [42]. A comprehensive understanding of the interplay between the gut microbiota and host health is imperative for the formulation of strategies aimed at preserving gut eubiosis, averting disease onset, and investigating microbiome-based therapeutic modalities [43].

The gut microbiota are integral to the maintenance of gynecological health, exerting significant influence on the pathophysiology of diverse gynecological disorders through its effects on immune modulation, hormone metabolism, and the inflammatory response [17]. Emerging research indicates that changes in the composition of the gut microbiota, referred to as dysbiosis, are implicated in various conditions, including PCOS, EM, bacterial vaginosis, and even gynecological malignancies [43]. The gut microbiota regulate estrogen metabolism through the estrobolome, a consortium of microbial genes that facilitate estrogen processing, thereby impacting estrogen-dependent disorders such as EM and hormone-related cancers [9,44]. Furthermore, the gut microbiota play a pivotal role in modulating systemic inflammation and immune responses, potentially aggravating chronic inflammatory conditions such as pelvic inflammatory disease (PID) and EM [22,25]. Research has also revealed a bidirectional relationship between the microbiota of the gut and the reproductive tract, indicating that gut dysbiosis may alter the microbial composition of the vaginal and cervical environments, thereby further influencing disease progression [27]. Therefore, elucidating the complex interplay between the gut microbiota and gynecological diseases may pave the way for microbiome-targeted therapeutic modalities, including probiotics, dietary modifications, and microbial modulation strategies, aimed at enhancing reproductive health and optimizing disease management.

Previous investigations examining alterations in the gut microbiota among individuals diagnosed with EM have elucidated significant modifications in the microbial diversity and composition (Table 1). Both alpha (*α*) and beta (*β*) diversities of the gut microbiota were observed to be markedly diminished in EM patients, indicating a comprehensive reduction in microbial richness and variability. α diversity, which assesses the variety and distribution of microbial species in a single sample, acts as a crucial sign of gut microbiome health and stability. A decrease in the *α* diversity, consistently observed in EM patients, signifies lower microbial diversity and reduced colonization resistance, potentially leading to inflammation and immune imbalance. In comparison, *β* diversity indicates the level of difference among microbial communities between various individuals or groups. A significant reduction in the *β* diversity within EM groups indicates a shift towards a more consistent microbial makeup, potentially resulting from immune responses linked to disease or environmental influences [42,43,45]. These alterations suggest a disturbance in the microbial balance that could be associated with the EM pathophysiology. Moreover, changes in certain microbial groups, such as a rise in *Bacteroides*, *Parabacteroides*, and *Oscillospira*, coupled with a reduction in helpful genera like *Lachnospira* and *Turicibacter*, reflect the noted diversity changes and highlight a wider dysbiotic pattern [45]. These microbial alterations become increasingly evident in later stages (III/IV), as indicated by additional decreases in *Sneathia, Barnesella*, and *Gardnerella* [46]. These results emphasize the capability of alpha and beta diversity metrics to serve as biomarkers for disease progression. Moreover, a higher Firmicutes/Bacteroidetes ratio and lower counts of helpful genera like Lachnospiraceae and Ruminococcaceae further demonstrate a pro-inflammatory microbiota configuration in EM [47,48]. Consequently, the noted decreases in diversity and shifts in composition indicate a considerable restructuring of the gut microbiome, potentially impacting systemic immunity and hormone-related pathways associated with disease progression.

**Table 1:** Features of gut microbiota changes in humans with EM in previous studies.

Research Type	Research Goal	Detection Technique	Results	Ref.
Case-control study	To investigate the association between gut microbiota and EM by comparing microbiota composition between women with EM and matched controls.	16S rRNA sequencing for bacterial identification at the genus level; statistical analyses including Mann–Whitney U test, Fisher’s exact test, Shannon diversity index, and Bray–Curtis dissimilarity index.	1. Overall gut microbiota diversity was significantly higher in controls than in EM patients. 2. Twelve bacterial genera from the Bacilli, Bacteroidia, Clostridia, Coriobacteriia, and Gammaproteobacteria classes differed significantly between groups before false discovery rate (FDR) adjustment. However, no significant differences remained within the EM group after FDR correction.	[45]
Prospective cohort study	To compare vaginal, cervical, and gut microbiota in women with stage 3/4 EM and healthy controls.	16S rRNA sequencing of the V3-V4 region; bioinformatics analysis including Shannon diversity index, Bray–Curtis dissimilarity index, and Wilcoxon tests.	1. *Atopobium* was absent in the vaginal and cervical microbiota of EM patients. 2. *Gardnerella, Streptococcus, Escherichia, Shigella,* and *Ureaplasma* were elevated in the cervical microbiota of the EM group. 3. More EM patients had *Escherichia/Shigella*-dominant stool microbiota.	[46]
Case-control study	To investigate the differences in gut microbiota composition between patients with stage 3/4 EM and healthy controls, and to analyze its associations with serum hormone levels and inflammatory factors.	16S rRNA high-throughput sequencing; PCR amplification of V3-V4 regions; Bio-Plex Pro Human Cytokine Panel for inflammatory factor analysis; SPSS 21.0 statistical analysis.	1. The EM group had lower α diversity, a higher Firmicutes/Bacteroidetes ratio, and distinct shifts in key taxa. 2. The EM group had *Prevotella_7* as the most abundant taxon. 3. The EM group exhibited significantly elevated estradiol levels (74.7 ± 22.5 pg/L vs. 47.9 ± 12.5 pg/L in controls) and IL-8 levels (6.39 ± 1.59 pg/mL vs. 4.14 ± 0.73 pg/mL in controls). 4. The EM gut microbiota was enriched in pathways for signaling, hormones, and immune modulation. 5. *Blautia* and *Dorea* were positively correlated with estradiol, while *Subdoligranulum* was negatively correlated with interleukin-8 (IL-8) levels.	[47]
Observational cohort study	To investigate microbial composition differences in gut, cervical mucus, and peritoneal fluid in EM patients and controls.	16S rRNA gene sequencing (Ion Torrent S5 platform), Bioinformatics analysis, Shannon and Simpson indices for diversity assessment, Bray–Curtis and Binary–Jaccard dissimilarity analysis, Principal Coordinate Analysis (PCoA).	1. Microbial profiles differed across sites, with more pathogens in peritoneal fluid and fewer protective microbes in EM feces. 2. Gut microbiota showed better diagnostic value, with *Ruminococcus* and *Pseudomonas* as EM biomarkers. 3. Dysbiosis in EM included less *Ruminococcus* and more *Pseudomonas*. Pro-inflammatory cytokines and estrogen levels were linked to reduced SCFA-producing gut bacteria.	[48]

While these investigations have significantly contributed to the understanding of microbial shifts associated with EM, inconsistencies in the findings illuminate the intricate nature of gut microbiota dynamics and the challenges inherent in establishing a definitive microbial signature for the disease [42]. Variability in the diagnostic criteria, classification methodologies, and fecal microflora detection techniques contributes to discrepancies among the studies, highlighting the necessity for standardized methodologies [40]. Additionally, the gut microbiota are subject to a multitude of influences, including genetic predispositions, dietary patterns, age, and geographic variances, which may further modulate the microbial composition and obscure the study results. Despite these complexities, the accumulating body of evidence robustly supports the proposition that the gut microbiota are intricately intertwined with the pathogenesis of EM [45]. These studies have profoundly augmented the comprehension of the association between the gut microbiota and EM by establishing microbial dysbiosis as a potential contributing factor to the etiology of the disease. They have elucidated the role of specific bacterial taxa in processes such as inflammation, estrogen metabolism, and immune regulation—key mechanisms implicated in the progression of EM. Furthermore, the identified alterations in the gut microbiota composition suggest the potential for microbial biomarkers for EM diagnosis and therapeutic targets for disease management. Comprehensive large-scale, multi-center clinical investigations employing standardized methodologies are imperative to validate these findings and ascertain their clinical significance within the context of EM pathophysiology and treatment strategies.

Functional profiles of gut microbiomes in EM are notably enriched in pathways related to environmental information processing, endocrine system regulation, and immune system functions when compared to healthy gut microbiomes [47]. Furthermore, alterations in the gut microbiota composition in EM are closely linked to the upregulation of the retinoic acid-inducible gene I (RIG-I)-like receptor signaling pathway, which activates the transcription of NF-kB target genes. This activation is associated with key biological processes such as the inflammatory response (IL-8 and TNF-α), apoptosis (Bax and Fas), cell proliferation (epidermal growth factor), and angiogenesis (VEGF) [47]. Additionally, a metabolic analysis comparing EM and healthy mice indicates significant differences, including reduced levels of alpha-linolenic acid—positively correlated with *Helicobacter* and *Ruminococcus*—and increased concentrations of primary and secondary bile acids, such as chenodeoxycholic acid (CDCA), which is negatively correlated with *Blautia* abundance, and ursodeoxycholic acid (UDCA), known for its anti-inflammatory properties [48].

Although there is substantial evidence demonstrating gut microbiota alterations in EM, inconsistencies in the findings due to limited data make it challenging to establish a definitive gut microbiota profile associated with the disease. Moreover, beyond gut microbiota changes being a consequence of EM, research from mouse models suggests a reciprocal relationship, wherein microbial imbalances may actively contribute to the progression of EM. These findings highlight the need for further studies to clarify the bidirectional influence between the gut microbiota and EM pathophysiology.

## 4. The Role of Gut Microbiota in the Pathogenesis and Progression of EM

The possible mechanism of gut microbiota involvement in EM is complex and involves both detrimental and protective effects on disease progression. EM is a chronic, estrogen-dependent inflammatory disorder characterized by the presence of endometrial-like tissue outside the uterus, leading to pain, infertility, and systemic inflammation [49]. Emerging evidence suggests that alterations in the gut microbiota may play a crucial role in the onset and progression of EM by influencing immune regulation, inflammation, and metabolic pathways [37].

Studies indicate that gut microbiota profiles could serve as diagnostic markers for EM, potentially surpassing the diagnostic capability of cervical microbiota [44]. This is largely due to the significant metabolic and inflammatory changes that occur as a result of gut microbiome dysbiosis, which can influence disease development both within and beyond the intestinal tract. Despite the field being in its early stages, strong associations between gut microbiota alterations and EM have been identified in human studies and animal models [44,45,46,47].

**Figure 2:**
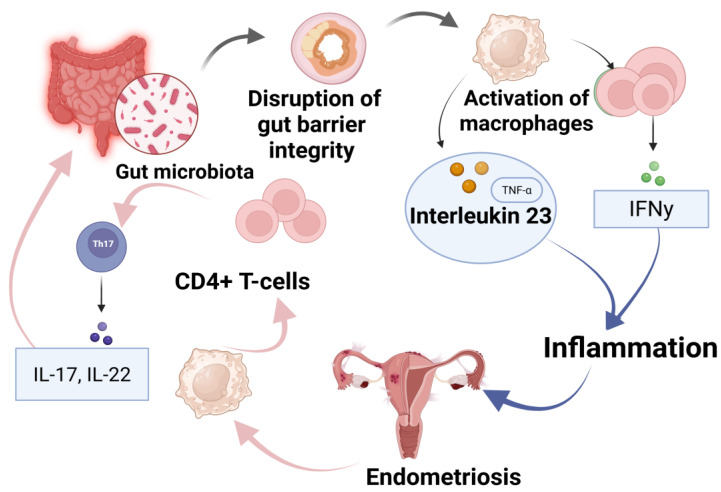
Immuno-microbial interactions in EM progression: the gut–immune–endometriosis axis and microbial metabolite influence [17,49]. Schematic representation of the proposed gut–immune–endometriosis axis. Gut microbiota dysbiosis leads to disruption of gut barrier integrity, activating immune responses including macrophages and CD4^+^ T-cells. This results in increased levels of interleukin-23 (IL-23) and TNF-α, stimulating Th17 cells to secrete pro-inflammatory cytokines IL-17 and IL-22. IFN-γ production further contributes to systemic inflammation, promoting endometriosis development and progression.

One proposed mechanism involves gut dysbiosis leading to a compromised gut barrier, allowing microbial metabolites and endotoxins to enter systemic circulation [49]. Gut microbiota alterations lead to the disruption of gut barrier integrity, allowing microbial components to enter systemic circulation, and subsequently triggering macrophages’ activation (Figure 2). These immune cells release pro-inflammatory cytokines, including TNF-α and interleukin-23 (IL-23), thereby promoting a persistent inflammatory response. Furthermore, CD4^+^ T-cells, particularly Th17 cells, contribute to this process by producing IL-17 and IL-22, which further exacerbate inflammation and create conditions conducive to endometriotic lesion survival and proliferation. As a result, EM lesions perpetuate this cycle by continuously stimulating immune cells, leading to systemic inflammation and disease progression. On the other hand, commensal gut microbiota play a protective role by producing SCFAs, which modulate immune responses via G-protein-coupled receptors (GPCRs) and inhibit histone deacetylases (HDACs), ultimately reducing inflammation and preventing lesion expansion [17,49]. Understanding these interactions is crucial for developing microbiota-targeted therapies for EM treatment and prevention.

The intestine represents one of the most immunologically dynamic organs within the human body, primarily attributable to its persistent exposure to an extensive array of antigens originating from consumed food, resident commensal microorganisms, and potential pathogenic entities [50]. In order to effectively manage this ongoing antigenic challenge, the gastrointestinal-associated lymphoid tissue (GALT) assumes a pivotal role in orchestrating immune responses [51]. The GALT comprises specialized lymphoid structures, encompassing Peyer’s patches, isolated lymphoid follicles, and mesenteric lymph nodes, which are instrumental in the recognition, processing, and response to various antigens [52]. This intricate immune network facilitates the activation of adaptive immune responses, thereby ensuring the proficient eradication of pathogens while concurrently preserving tolerance towards commensal microbiota and dietary antigens [53].

The gut microbiota possesses a substantial immunomodulatory capacity, significantly affecting both innate and adaptive immune mechanisms [43]. It contributes to the maintenance of immune homeostasis by fostering the maturation and functionality of immune cells, reinforcing mucosal barrier integrity, and modulating inflammatory pathways [51,52,53,54]. Through their interactions with intestinal epithelial cells (IECs) and immune cells, the gut microbiota assist in sustaining the stability of the intestinal microenvironment by inhibiting the proliferation of pathogenic bacteria, fine-tuning immune responses, and metabolizing deleterious substances [51]. Moreover, microbial metabolites, including SCFAs, secondary bile acids, and indole derivatives, play crucial roles in immune regulation by modulating cytokine synthesis, influencing the differentiation of regulatory T cells (Tregs), and enhancing epithelial barrier function [55].

A substantial array of microorganisms colonizes the intestinal mucosa, providing pathogen-associated molecular patterns (PAMPs) that significantly contribute to host immune modulation. These PAMPs encompass lipopolysaccharides (LPS), peptidoglycans, flagellin, and lipoproteins, which interact with pattern recognition receptors (PRRs) such as Toll-like receptors (TLRs) that are prevalently expressed on IECs and immune cells [56]. The interaction of PAMPs with PRRs initiates downstream signaling cascades that stimulate the secretion of pro-inflammatory chemokines (e.g., IL-8), cytokines (e.g., IL-1, IL-6, IL-7, IL-11, and TNF-α), and growth factors (e.g., stem cell factor (SCF) and granulocyte colony-stimulating factor (G-CSF)). Collectively, these molecular entities contribute to the recruitment of peripheral neutrophils and mast cells to the intestinal mucosa, thereby facilitating the activation and differentiation of local lymphocytes [57,58,59].

Despite extensive investigations, the precise molecular mechanisms governing the interactions between the microbiota and the immune system remain inadequately elucidated. Additional research is imperative to clarify the intricate signaling pathways through which the gut microbiota exert influence on immune responses and to explore potential therapeutic approaches aimed at targeting microbiota-immune interactions for the prevention and management of immune-related disorders [60].

Gut dysbiosis and bacterial metabolites play a crucial role in compromising intestinal barrier integrity, facilitating the translocation of bacteria and endotoxins into systemic circulation. This process disrupts immune homeostasis, leading to oxidative stress and chronic inflammation, thereby contributing to the development and progression of various autoimmune diseases [61]. Researchers have increasingly explored the potential link between autoimmune diseases and EM, as EM shares several immunopathological features with autoimmune conditions, including polyclonal B cell activation, dysregulated T and B cell function, impaired apoptosis, tissue damage, and multi-organ involvement [62,63,64]. Moreover, EM frequently coexists with autoimmune diseases, further supporting the hypothesis of immune dysregulation as a key pathogenic mechanism.

The gut microbiota play a critical role in shaping immune responses by influencing the differentiation of T lymphocytes into distinct subsets, including helper T cells (Th1, Th2, and Th17) and regulatory Tregs [65]. Specific microbial taxa have been implicated in modulating immune responses, such as segmented filamentous bacteria (SFB) directly stimulating Th17 differentiation, *Clostridium* spp. promoting Treg development, and *Bacteroides* regulating the balance between Th1 and Th2 cells. Studies have demonstrated that Th17 cells and their associated cytokine profiles are significantly elevated in the peritoneal fluid of individuals with EM, with excessive IL-17 production correlating with the disease severity [66]. Additionally, research comparing the gut microbiota diversity between EM patients and healthy controls has identified reduced *α*-diversity and an increased *Firmicutes/Bacteroidetes* ratio, which is a hallmark of dysbiosis [67]. Furthermore, IL-17A levels were found to be significantly reduced in EM patients and negatively correlated with the abundances of *Streptococcus* and *Bifidobacterium*, suggesting a complex interplay between the gut microbial composition and immune dysregulation [68,69].

Polysaccharides are a key component of the outer membrane of Gram-negative bacteria, known for their strong immunogenic properties that trigger host immune responses (Table 2). In gut dysbiosis, an overgrowth of Gram-negative bacteria leads to increased intestinal permeability, allowing LPS leakage into systemic circulation [70]. This process disrupts immune homeostasis by activating Toll-like receptor 4 (TLR4), which stimulates the nuclear factor kappa-light-chain-enhancer of activated B cells (NF-κB) signaling and promotes inflammation through cytokine production, including TNF-*α*, IL-8, and IL-6 [71]. Elevated LPS levels in the peritoneal fluid of patients with EM suggest its role in the chronic inflammation associated with the disease [72]. Moreover, LPS has been shown to enhance the proliferation of endometrial stromal cells, contributing to lesion formation [73].

In contrast, SCFAs, such as acetate, propionate, and butyrate, are fermentation by-products of the gut microbiota that play a crucial role in maintaining gut integrity, modulating immune responses, and exerting anti-inflammatory effects [74]. SCFAs regulate immune signaling by activating GPCRs and inhibiting HDACs, thereby reducing the inflammatory response [75]. Studies indicate that patients with EM exhibit decreased SCFA levels, particularly butyrate, which has been associated with compromised immune regulation and enhanced lesion growth. An SCFA deficiency may contribute to increased inflammation by promoting the activation of the NOD-like receptor pyrin domain-containing 3 (NLRP3) inflammasome, further linking gut dysbiosis to EM pathogenesis [76,77,78]. These findings suggest that targeting LPS-induced inflammation and restoring SCFA levels could be promising therapeutic approaches for managing EM.

**Table 2:** The role of LPS and SCFAs in gut microbiota and EM [70,71,72,73,74,75,76,77,78].

Factor	Source	Mechanism of Action	Impact on EM	Potential Therapeutic Implications	References
LPS	Gram-negative bacteria (e.g., *Pseudomonadaceae Pseudomonas*, *Prevotellaceae*, *Prevotella*)	Binds to Toll-like receptor 4 (TLR4), activates NF-κB via MyD88-dependent and independent pathways, promotes pro-inflammatory cytokine secretion (TNF-α, IL-8, IL-6)	Increases inflammation, enhances peritoneal macrophage activation, promotes endometriotic lesion growth, disrupts immune homeostasis	Blocking TLR4/NF-κB signaling, anti-TNF-α therapy, probiotics to reduce Gram-negative bacteria	[70,71,72,73]
SCFAs (acetate, propionate, butyrate)	Produced by gut microbiota fermentation of dietary fibers (*Lachnospiraceae Ruminococcus*, *Clostridium* spp.)	Activates GPCRs (GPR41, GPR43, GPR109A), inhibits histone deacetylases (HDACs), modulates immune response, suppresses pro-inflammatory cytokines (TNF-α, IL-6)	Decreased SCFA levels in EM patients, reduced inhibition of NF-κB, enhanced NLRP3 inflammasome activation, increased histone deacetylation (HDAC-1) promoting lesion growth	Probiotics to restore SCFA-producing bacteria, butyrate supplementation, HDAC inhibitors to modulate gene expression	[74,75,76,77,78]

The gut microbiota play a crucial role in modulating metabolic pathways and immune responses, which can lead to localized and systemic inflammatory conditions [79]. This persistent low-grade inflammation is a key feature of EM, a chronic estrogen-dependent inflammatory disorder [80]. Emerging research has linked gut microbiota dysbiosis to the progression of EM, demonstrating that alterations in the microbial composition can influence immune regulation and inflammatory signaling [81]. For instance, studies have shown that treating mice with broad-spectrum antibiotics or metronidazole resulted in significantly smaller endometriotic lesions and reduced levels of inflammatory cytokines such as IL-1*β*, TNF-*α*, IL-6, and TGF-*β*1 in the peritoneal fluid [82]. The inhibitory effect of metronidazole on bacteroides growth appears to play a role in reducing lesion formation. Fecal Microbiota Transplantation (FMT) is also being explored as a method to restore gut microbial balance in inflammatory disorders. Furthermore, FMT from EM-induced mice into antibiotic-treated mice restored lesion growth and inflammation, suggesting a direct role of the gut microbiota in EM pathogenesis [83].

Microbial composition studies have identified elevated levels of *Streptococcus* in patients with advanced-stage EM (stage III/IV), correlating with increased IL-8 serum levels [84]. Previous research has demonstrated that *S. bovis* induces the overexpression of NF-κB, IL-1, IL-8, and cyclooxygenase-2 (COX-2), a key enzyme in the synthesis of prostaglandin E2 (PGE2), which is associated with chronic pelvic pain in EM [85]. Given the role of COX-2 in promoting inflammation and lesion survival, COX-2 inhibitors have been proposed as a potential therapeutic approach for reducing EM-associated pain and inflammation. Additionally, *Ruminococcaceae*, a bacterial family negatively associated with intestinal epithelial apoptosis and IL-6 levels, was found to be significantly reduced in EM patients [86]. This suggests that a decrease in *Ruminococcaceae* may exacerbate pelvic inflammation and contribute to disease progression.

The gut microbiota composition in patients with EM exhibits significant alterations, with an increase in pro-inflammatory bacterial taxa such as *Streptococcus* and *Escherichia coli*, alongside a reduction in beneficial bacteria like *Ruminococcaceae*, which are known to regulate immune responses and maintain intestinal homeostasis (Table 3) [87,88,89,90,91,92,93,94,95].

Another notable factor is the presence of *Escherichia coli* (*E. coli*) in menstrual blood of EM patients, which can reflux into the pelvic cavity, leading to elevated endotoxin (LPS) levels in peritoneal fluid [87]. LPS activates TLRs, specifically TLR4, triggering a cascade of pro-inflammatory cytokine production, including TNF-*α*, IL-6, and IL-8 [88]. This inflammatory microenvironment enhances adhesion molecule expression and facilitates the implantation and growth of ectopic endometrial cells. Such microbial-induced inflammatory responses may drive EM lesion formation through ectopic adhesion, invasion, and proliferation [43].

Although the precise pathological mechanisms of EM remain unclear, extensive epidemiological and clinical studies confirm that estrogen plays a pivotal role in disease progression [87]. Estrogen promotes cell adhesion, invasion, the proliferation of ectopic lesions, and inflammation, while also inhibiting apoptosis [89]. The biological effects of estrogen are mediated through its receptors, estrogen receptor alpha (ER*α*), and estrogen receptor beta (ER*β*), as well as the G protein-coupled estrogen receptor (GPER1). These receptors interact with estrogen response elements (EREs) or other transcription factors such as NF-κB, activator protein-1 (AP-1), and stimulating protein-1 (SP-1) to regulate gene expression [90]. In addition to genomic estrogen signaling, non-genomic pathways involving mitogen-activated protein kinase (MAPK), phosphoinositide 3-kinase (PI3K), and cyclic adenosine monophosphate (cAMP) also contribute to EM pathogenesis, although their precise roles require further investigation [91].

Recent studies suggest that the gut microbiota can modulate estrogen metabolism, influencing the risk of estrogen-dependent diseases [92]. The primary sources of estrogen in the body include the ovaries, adrenal glands, and adipose tissue, after which estrogen undergoes hepatic metabolism and conjugation [52]. Gut microbiota, particularly those producing *β*-glucuronidase, can deconjugate estrogen metabolites, increasing their reabsorption via enterohepatic circulation [93]. This process significantly elevates systemic estrogen levels, stimulating the growth of ectopic lesions and promoting periodic bleeding in EM. Previous studies have identified *Clostridia* and *Ruminococcaceae* spp. as key contributors to estrogen activation, further supporting their role in estrogen-dependent diseases [91,92,93,94].

Conversely, *Lactobacillus* and *Bifidobacterium* have been found to reduce *β*-glucuronidase-producing bacteria, thereby decreasing estrogen reabsorption and potentially mitigating EM progression [95]. A study by Shan et al. found that patients with advanced EM exhibited significantly higher levels of *Blautia* and *Dorea*, both of which positively correlated with systemic estrogen levels [46]. However, the exact mechanisms linking the gut microbiota, estrogen metabolism, and EM remain to be elucidated. Future research should focus on identifying microbial biomarkers and microbiota-targeted interventions, such as probiotics, prebiotics, and FMT, to modulate the gut microbiota and disrupt the estrogen–microbiota–inflammation cycle driving EM progression [95].

An increasing amount of epidemiological research indicates that EM is often linked to various autoimmune disorders, though the strength of these links differs and is often constrained by the quality of the existing data. A recent systematic review of 26 population-based cross-sectional, case–control, and cohort studies revealed that EM was most frequently associated with systemic lupus erythematosus, Sjögren’s syndrome, rheumatoid arthritis, autoimmune thyroid disease, celiac disease, multiple sclerosis, inflammatory bowel disease, and Addison’s disease [96]. Nonetheless, the evidence quality backing these associations was generally rated as ‘low’ or ‘very low’ according to GRADE standards, primarily due to methodological issues like limited sample sizes, inadequate adjustment for confounding variables, and ambiguous temporal relationships among diagnoses. Merely four high-quality studies offered strong evidence for a notable link between EM and autoimmune diseases, particularly systemic lupus erythematosus, Sjögren’s syndrome, rheumatoid arthritis, celiac disease, multiple sclerosis, and inflammatory bowel disease [97,98,99,100]. Although the evidence is somewhat weak, these results indicate a non-random co-occurrence pattern that requires additional investigation via well-structured prospective studies involving large populations and strict disease classification.

The presence of endometriosis alongside specific autoimmune diseases might indicate common immunopathogenic mechanisms, although definitive causality has yet to be confirmed. Immunological irregularities noted in women with EM include heightened peritoneal levels of neutrophils and macrophages, the reduced cytotoxic function of natural killer cells, and unregulated populations of T and B lymphocytes, all of which contribute to an inflammatory microenvironment that may promote autoimmunity [101,102]. Moreover, increased concentrations of different autoantibodies have been detected in EM patients, suggesting that certain autoimmune reactions might contribute to disease onset or advancement [103,104]. Additionally, genetic research has linked various autoimmune-related genes, including PTPN22 and specific HLA alleles, to the development of EM, though results are still ambiguous because of the small sample sizes and restricted genomic coverage [105,106]. Hormonal factors may serve as a cohesive element, as estrogen is recognized for its ability to adjust immune functions and intensify autoimmune responses. For example, estrogen-stimulating assisted reproductive therapies have been linked to a heightened risk of worsening multiple sclerosis, emphasizing the intricate relationship between hormonal control and immune dysfunction in women with EM [107].

## 5. Key Discoveries Linking EM and Malignant Transformation

**Figure 3:**
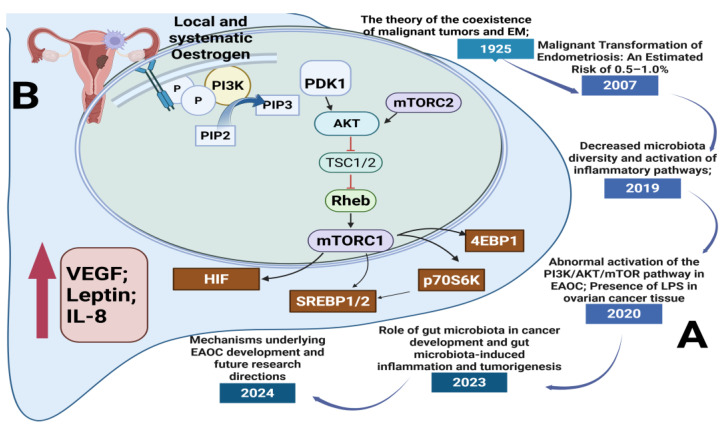
Timeline of key discoveries and molecular mechanisms linking EM to malignant transformation via the mTOR pathway: (A) Historical and scientific milestones: significant findings from 1925 to 2024 linking endometriosis (EM), alterations in gut microbiota, and malignant transformation; (B) Cellular/molecular mechanisms (PI3K/AKT/mTOR pathway involvement in EAOC): the PI3K/AKT/mTOR pathway implicated in EAOC). Estrogen stimulates PI3K signaling, resulting in mTORC1 activation and its downstream targets (4EBP1, p70S6K, SREBP1/2, HIF), enhancing cell growth, angiogenesis, and inflammation. Cytokines such as VEGF, IL-8, and leptin play a role in creating an environment that promotes tumor growth [12,112,113,114,115,116,117,118,119,120,121,122,123,124].

The potential association between EM and malignant transformation was first proposed following the observation of malignant tumors coexisting with EM within the same lesion, as reported in 1925 by Sampson (Figure 3). This observation was based on a histopathological analysis of ovarian tumors in patients with a history of EM. Sampson’s findings were influenced by the growing recognition of chronic inflammation as a contributing factor to tumorigenesis and the observation that some endometriotic lesions displayed cellular atypia and invasive properties, resembling early-stage malignancies. His hypothesis proposed that persistent estrogen stimulation, chronic inflammatory responses, and genetic alterations might contribute to the malignant transformation of endometriotic cells [12,18,32].

Under specific conditions or stimuli, EM has the potential to undergo malignant transformation, with an estimated transformation rate of approximately 0.5–1.0%. This estimation was derived from epidemiological studies and retrospective analyses of histopathological samples from patients diagnosed with ovarian cancer and concurrent EM. Researchers identified a progression from benign endometriotic lesions to atypical EM and ultimately to invasive carcinoma [12,108,109]. However, further large-scale cohort studies and molecular analyses are needed to better understand the mechanisms underlying this transformation. The advanced genomic and transcriptomic profiling of endometriotic lesions could help identify key oncogenic pathways, genetic mutations, and epigenetic alterations associated with EM-associated ovarian cancer (EAOC) development [110]. Additionally, longitudinal studies tracking patients with EM over time may provide insights into predictive biomarkers for malignant transformation, allowing for earlier detection and intervention. This transformation predominantly occurs in the ovaries, where EAOC is characterized by the presence of cancerous tissue adjacent to ectopic endometrial lesions, exhibiting a continuum of changes from the ectopic endometrium to atypical hyperplasia and malignancy. It is widely recognized that ovarian clear cell carcinoma (CCC) and ovarian endometrioid carcinoma (OEC) originate from EM [111]. However, the precise molecular mechanisms underlying EAOC pathogenesis remain unclear.

The mechanistic target of the rapamycin (mTOR) signaling pathway is pivotal in the malignant transformation of EM, mainly via its control of cell growth, metabolism, proliferation, and autophagy. mTOR operates within two separate multiprotein complexes: mTORC1 and mTORC2. mTORC1 consists of mTOR, raptor, PRAS40, Deptor, and mLST8, whereas mTORC2 consists of mTOR, rictor, mSIN1, Protor-1, Deptor, and mLST8 [12,112]. The activation of mTORC1 takes place through two upstream signaling pathways: signaling from growth factors that trigger PI3K/AKT and the activation of Rag and Rheb GTPases induced by amino acids. When activated, mTORC1 moves to the lysosomal membrane, where Rheb directly enhances its kinase activity. This results in the phosphorylation of downstream targets like p70S6K, 4EBP1, and sterol regulatory element-binding proteins (SREBP1/2), which promotes increased protein and lipid biosynthesis, cell cycle advancement, and metabolic reprogramming. Simultaneously, mTORC1 function is regulated by PRAS40, which restrains the complex until it is phosphorylated by AKT, enabling substrate access. The TSC1/2 tumor suppressor complex inhibits mTORC1 by blocking Rheb, but the activation of AKT interferes with this complex, enhancing mTOR signaling. The absence of PTEN, which negatively regulates PI3K/AKT signaling, enhances this pathway, a characteristic often seen in EAOC. Additionally, mTORC1 suppresses autophagy by phosphorylating ULK1 in nutrient-abundant environments, whereas AMPK activation during energy shortages restarts autophagic activity. The dual function of autophagy in cancer introduces complexity, as it might aid in early tumor suppression while also enhancing survival during therapeutic stress. Significantly, mTOR activation promotes a transition from oxidative phosphorylation to glycolysis, enhancing GLUT1 and glycolytic enzymes such as HK2 to aid in swift cell proliferation. Moreover, the activation of SREBP1 by mTOR promotes de novo lipid creation, which further accelerates tumor growth. Chronic inflammation, ongoing estrogen exposure, and oxidative stress in EM foster a tumor-friendly microenvironment where mTOR-driven pathways remain continuously activated. The decline of Deptor, a natural inhibitor of mTOR, worsens pathway activation and is associated with a worse prognosis. Collectively, these mechanisms facilitate the change of non-cancerous endometriotic lesions into cancer, especially in the ovaries. Due to its diverse functions, the mTOR pathway serves as a crucial therapeutic target and a valuable area for biomarker advancement in the early identification and management of EM-related cancers [12,112,113,114,115,116,117,118,119,120,121,122,123,124].

A growing body of research supports the notion that chronic inflammation is a fundamental process in tumor development, with inflammatory diseases and infections contributing to approximately 25% of cancers worldwide [112]. Recent studies highlight the critical role of gut microbiota in cancer development [113,114,115,116,117,118,119,120,121,122,123,124,125]. Gut microbes can upregulate TLRs and activate NF-κB, thereby inducing the secretion of pro-inflammatory cytokines such as IL-6, IL-12, IL-17, IL-18, and TNF-α [117]. These cytokines create a persistent inflammatory microenvironment conducive to tumorigenesis [118,119]. It was found that microbial diversity and richness were significantly reduced in ovarian cancer tissues compared to healthy distal fallopian tube tissues. Additionally, it was observed that inflammation-associated pathways, including NF-κB signaling, cytokine–cytokine receptor interactions, and chemokine signaling pathways, were markedly activated in ovarian cancer samples [120].

Additionally, research has identified the presence of LPS in cancerous ovarian tissues [121]. LPS, a component of the outer membrane of Gram-negative bacteria, interacts with TLR4, leading to the activation of downstream signaling pathways such as PI3K/AKT [57]. This interaction promotes the phosphorylation of AKT, ultimately facilitating cellular proliferation, invasion, and metastasis [122]. The dysregulation of the PI3K/AKT/mTOR pathway has been extensively reported in various malignancies, including EAOC, and is considered a potential therapeutic biomarker for CCC [123,124]. These findings suggest that the gut microbiota may contribute to the initiation and progression of ovarian cancer by modulating local inflammatory responses or by activating oncogenic signaling pathways.

Furthermore, elevated estrogen levels have been identified as a significant risk factor for the malignant transformation of EM. Estrogen dysregulation can promote the hyperplasia and malignancy of ectopic endometrial tissue by increasing aromatase expression and enzymatic activity. As discussed previously, gut dysbiosis plays a pivotal role in estrogen metabolism by increasing systemic estrogen levels through enterohepatic circulation. This, in turn, fosters a pro-inflammatory microenvironment that may synergistically drive carcinogenesis when combined with hormonal dysregulation within endometriotic lesions [125]

Given these insights, it is plausible that the gut microbiota are intricately involved in the malignant transformation of EM. However, the precise mechanisms by which the gut microbiota influence this transformation remain largely unknown. Further research is warranted to elucidate whether the regulation of the gut microbiota could serve as a preventive or therapeutic approach to mitigate the risk of EM-associated malignancies. Future investigations should focus on identifying microbial biomarkers, exploring targeted microbiome interventions, and assessing the therapeutic potential of modulating the gut microbiota in the prevention and treatment of EAOC.

## 6. Diagnosis and Treatment

The delayed diagnosis of EM remains a significant clinical challenge, with patients often experiencing prolonged incubation periods before receiving a confirmed diagnosis. The time delay, which can range from 4 to 11 years, is primarily due to the nonspecific early symptoms and the necessity of invasive diagnostic techniques [126]. Laparoscopic surgery with histological confirmation remains the gold standard for diagnosing EM; however, its invasive nature makes it an impractical routine screening method for all suspected cases [127]. Consequently, there is an urgent need to identify minimally invasive or non-invasive biomarkers for early detection.

With the growing body of research on the gut microbiome in EM, specific gut microbial alterations have emerged as potential diagnostic markers [17]. Several studies have identified gut dysbiosis in both women and murine models with EM, providing insight into potential microbial biomarkers. It was found that the depletion of *Lachnospiraceae Ruminococcus* in the feces of EM patients could serve as a diagnostic indicator [47]. Moreover, Ata et al. found that two out of fourteen patients with stage III/IV EM had gut microbiota dominated by *Escherichia* and *Shigella* [45]. Notably, these patients exhibited severe gastrointestinal involvement, ultimately requiring a segmental colectomy, whereas the controls exhibited no similar microbial patterns. This finding underscores the potential for gut microbiome analysis in predicting severe bowel involvement in EM.

Additionally, a two-sample Mendelian randomization (MR) study identified eight gut microbiota taxa significantly associated with EM. Among them, Class-Melainabacteria, Family-*Ruminococcaceae*, and Genus-*Eubacterium ruminantium* exhibited a protective role against EM. Conversely, Order-*Bacillales*, Family-*Prevotellaceae*, Genus-*Anaerotruncus*, Genus-*Olsenella*, and Genus-*Ruminococcaceae* UCG002 were linked to an increased risk of EM [128].

Beyond the microbial composition, gut microbiota-derived metabolites have gained attention as potential diagnostic and therapeutic targets. It was identified that quinic acid was significantly upregulated in the feces of EM mice, which has been shown to promote cellular proliferation and lesion growth. Similarly, another study identified four key metabolites such as CDCA, UDCA, ALA, and 12,13-epoxy-9z,11,15z octadecatrienoic acid (12,13-EOTrE), which have potential non-invasive diagnostic applications in EM [129].

The current treatment strategies for EM primarily rely on medical therapies that suppress the ovarian function or surgical excision of endometriotic lesions [130]. First-line pharmacological interventions include nonsteroidal anti-inflammatory drugs (NSAIDs), oral contraceptives, progestins, gonadotropin-releasing hormone (GnRH) analogs, aromatase inhibitors, and androgen analogs. However, these approaches are not curative and are frequently associated with recurrence and adverse side effects [131]. Given the high recurrence rate (40–50%) following surgical resection, alternative therapies targeting the gut microbiome have garnered increasing interest [132].

Probiotics, defined as live microorganisms that confer health benefits, have been extensively investigated for their role in modulating the gut microbiota composition, immune function, and intestinal barrier integrity [133]. The two most studied probiotic genera, Lactobacillus and Bifidobacterium, have been shown to enhance mucosal immunity, inhibit the growth of pathogenic bacteria, and reduce inflammation [134].

Several studies suggest that probiotics may hold therapeutic potential in EM [109]. For instance, a study related to probiotics demonstrated that *Lactobacillus gasseri* OLL2809 inhibited EM lesion growth via the activation of natural killer (NK) cells in mice [134]. Moreover, a randomized, double-blind, placebo-controlled trial found that the oral administration of *L. gasseri* OLL2809 significantly reduced menstrual pain and dysmenorrhea in women with EM, with no reported adverse effects [135]. Additionally, a pilot triple-blind, placebo-controlled trial was conducted, indicating that oral *Lactobacillus* supplementation significantly alleviated EM-associated pain.

Diet plays a pivotal role in shaping the gut microbiota composition, making dietary modifications an attractive therapeutic strategy for EM [124]. Among the most studied dietary components, omega-3 polyunsaturated fatty acids (PUFAs) have demonstrated anti-inflammatory and immunomodulatory properties [136,137]. Omega-3 PUFAs, including docosahexaenoic acid (DHA), eicosapentaenoic acid (EPA), ALA, and docosapentaenoic acid (DPA), are known to reduce inflammatory cytokines such as TNF-α, IL-1β, and IL-6, thereby mitigating chronic inflammation in EM [138].

Notably, animal studies have shown that omega-3 PUFA supplementation suppressed endometriotic lesion growth [139]. Furthermore, a prospective cohort study of premenopausal women reported that long-term omega-3 PUFA consumption significantly reduced the incidence of EM [140]. Additionally, ALA supplementation has been shown to restore beneficial gut microbiota composition, decrease LPS levels in the peritoneal cavity, and reduce inflammatory macrophage aggregation in EM mice [141,142]. Collectively, these findings suggest that omega-3 PUFAs could serve as a promising dietary intervention for EM prevention and treatment.

The low-FODMAP diet (Fermentable Oligo-, Di-, Monosaccharides, And Polyols diet) has shown potential in modulating the gut microbiota in patients with endometriosis [142]. A retrospective analysis found that a low-FODMAP diet alleviated bowel symptoms in women with EM, potentially via gut microbiota-related pathways [143]. However, some studies have reported that low-FODMAP diets reduce beneficial gut bacteria, including *Bifidobacterium* and *Faecalibacterium prausnitzii*, and decrease SCFAs, which are essential for gut health [144]. Thus, the selective reintroduction of restricted foods is recommended following a dietary intervention. Despite its potential, further research is needed to confirm the efficacy of dietary modifications in EM management [145,146].

FMT, which involves the transfer of fecal microbiota from healthy donors to individuals with dysbiosis, has emerged as a novel therapeutic approach for gut microbiota-mediated diseases [147]. It has been successfully used to treat *Clostridium difficile* infections (CDI), with an efficacy rate exceeding 90% [148]. Moreover, FMT has shown promise in managing ulcerative colitis, irritable bowel syndrome, depression, and metabolic syndrome [149,150,151,152,153]. However, FMT has not yet been explored for EM treatment, necessitating further preclinical and clinical investigations to determine its feasibility.

As our understanding of the gut microbiota–EM axis expands, novel diagnostic and therapeutic strategies are emerging. Microbiota-based interventions, including probiotics, dietary modifications, and FMT, may offer promising alternatives to conventional EM treatments. Future research should focus on validating microbial biomarkers, elucidating microbial–metabolite interactions, and assessing microbiota-targeted therapies to improve EM diagnosis and management.

The idea of therapeutic windows in treating EM is still unclear, as there is no defined timeframe when treatment effectively stops disease progression or recurrence [154]. In contrast to cancer, which generally shows improved results with early treatment, EM does not have a specific “window of opportunity” for successful intervention [143,154]. There are no established preventive medical therapies, and the efficacy of adjuvant treatment after surgery is still unclear. Furthermore, managing recurrence poses a major challenge due to the absence of dependable, non-invasive diagnostic tools for tracking disease advancement [153]. The lack of agreement on defining recurrence—whether through symptoms, imaging, or surgical results—adds to the complexity of treatment strategies. A life-course impact evaluation has highlighted the complex burden of EM, underscoring the necessity for timely detection and customized management approaches suited to various life stages [149]. This underscores the need for innovative methods, like improved biomarkers for early detection and targeted treatments that could potentially alter disease progression instead of just relieving symptoms.

The surgical treatment of EM is commonly used, especially with laparoscopic excision, yet its long-term effectiveness is still a topic of discussion (Table 4) [154]. Current global guidelines suggest the surgical excision of all types of EM, as the evidence indicates that it alleviates pain more effectively than diagnostic laparoscopy by itself. Nevertheless, the backing data are derived from a restricted number of randomized controlled trials (RCTs) that feature small sample sizes, resulting in the evidence being of a moderate-to-low quality [155]. Although surgery might help with deep EM, its effectiveness for superficial peritoneal EM remains unclear, as there is no conclusive evidence that it enhances long-term quality of life. Furthermore, multiple surgeries could worsen symptoms instead of offering lasting relief. The surgical treatment of ovarian EM is especially intricate because of worries regarding the loss of ovarian reserves and its possible effects on fertility. There are no existing guidelines that specify a safe cyst size threshold for surgery in patients without symptoms. Therefore, well-conducted RCTs comparing various surgical methods, like cyst ablation and stripping, are crucial for identifying the best balance among symptom relief, fertility conservation, and recurrence prevention [156].

Ongoing research highlights the significant role of the gut microbiota in EM pathogenesis, diagnosis, and treatment [32]. While advancements in genome sequencing and microbiome-derived metabolite analysis have deepened our understanding, challenges remain in establishing causal relationships, refining diagnostic biomarkers, and integrating microbiota-based therapies into clinical practice (Table 5) [139,153]. Clearly, further large-scale studies and advancements in bioinformatics are necessary to overcome these limitations and develop more effective, personalized treatment strategies [151].

The usefulness of the summarized data in clinical settings has improved by organizing therapeutic gaps, evidence strength, and actionable suggestions in a structured manner (Table 4). Recent research emphasizes the significance of pinpointing an ideal intervention window in EM, which is still not clearly defined and poses an obstacle to prompt management and recurrence avoidance [157]. This issue is worsened by the lack of dependable monitoring methods, yet the advancement of blood- and urine-based biomarkers provides hopeful pathways for early identification and post-surgery monitoring [158]. The recent surgical literature indicates that the efficacy of interventions such as laparoscopic excision for superficial peritoneal and ovarian EM remains contentious, especially because of worries regarding long-term advantages and possible damage to ovarian reserves [159,160]. This highlights the importance of fertility-preserving approaches and personalized risk evaluation. Additionally, recent findings endorse the application of anti-Müllerian hormone testing and focused fertility guidance to enhance patient results before surgical procedures [161]. These evidence-based factors support the organized method of decision making emphasized in the dataset and strengthen the importance of the clinical implications and suggested actions included.

An increasing number of studies have elucidated the function of the gut microbiota in the development, diagnosis, and treatment possibilities of EM (Table 5). Recent integrative research has shown that microbial metabolites, including short-chain fatty acids and bile acid derivatives, can impact estrogen metabolism and inflammatory pathways essential to the progression of EM [162,163]. Moreover, progress in high-throughput sequencing, along with machine learning algorithms, has facilitated improved disease prediction and classification, thus providing a possible foundation for non-invasive diagnostic methods [164]. Early interventional studies indicate that treatments focused on altering the microbiota, such as probiotics and dietary changes, could alleviate symptoms and lower recurrence rates, thereby improving the quality of life for those impacted [165]. Moreover, studies on the interactions between the gut microbiota and drug metabolism offer fresh prospects for the customization of pharmacological therapies. This is especially crucial in hormone-sensitive disorders such as EM, where variations in metabolism among individuals can affect the effectiveness of treatment [166]. These findings, as shown in the outlined limitations and research paths, emphasize the practical importance of emerging microbiome science in bridging the translational gap between research and tailored clinical care approaches.

## 7. Potential Gut Microbiome-Based Therapies for EM

The diagnostic delay for endometriosis, typically lasting from 4 to 12 years, is mainly due to the vague nature of its symptoms and the dependence on invasive laparoscopic methods for verification [167]. Although imaging technologies like transvaginal ultrasound and magnetic resonance imaging have enhanced diagnostic precision, particularly for deep infiltrating endometriosis, challenges remain in identifying superficial lesions and in obtaining access to experienced specialists. As a result, microbiome testing is increasingly recognized as a non-invasive supplement for diagnosing endometriosis. Research indicates that unique microbial patterns might be present in women with endometriosis, possibly enabling classification according to the microbial composition from various body sites including feces, cervical mucus, and peritoneal fluid [168,169,170,171].

Khan et al. [172] observed a notable rise in *E. coli* levels in the menstrual blood of women who had both ovarian endometriomas and superficial peritoneal lesions, suggesting a possible connection between microbial dysbiosis and the pattern of lesions. Supporting evidence from Huang et al. [173] adds to the potential diagnostic value of microbial markers. In their research, samples obtained from feces, cervical mucus, and the peritoneal fluid of patients with endometriosis showed changes in the microbiota composition compared to healthy controls. Significantly, the microbiota in the gut and peritoneal fluid showed greater variations, characterized by a reduction in beneficial microbes in feces and a higher prevalence of pathogens in peritoneal fluid. Employing machine learning models, the research pinpointed *Ruminococcus* and *Pseudomonas* as potential diagnostic biomarkers, revealing that the gut microbiota serve as a more dependable diagnostic marker compared to cervical microbiota.

Aside from diagnostic uses, achieving a gut microbial equilibrium via targeted therapeutic methods is becoming acknowledged as a hopeful strategy for addressing inflammation and symptom burden in endometriosis. Antibiotics, FMT, dietary changes, and probiotic supplementation can be used to modify microbiota [174,175,176]. Among these choices, probiotics, known as live microorganisms that offer health benefits when given in adequate amounts, have gained significant interest for their capacity to influence immune responses and aid in the recovery of a healthy gut microbiota. Importantly, specific bacterial genera like *Akkermansia* have shown promise in reducing inflammation linked to gynecological conditions [177,178,179]. Nonetheless, further studies are required to verify their safety, efficacy, and suitable clinical uses regarding endometriosis [180].

Numerous studies have investigated the therapeutic capabilities of *Lactobacillus* species in influencing immune responses in individuals with endometriosis. A certain in vitro study indicated that *Lactobacillus acidophilus* affected cytokine production byPBMCs, initially boosting pro-inflammatory cytokines like IL-1 and IL-6, but subsequently reducing their levels after 48 h, suggesting a modulatory effect [181]. Additionally, a randomized, placebo-controlled pilot study conducted with women experiencing stage 3 and 4 endometriosis showed that 8 weeks of oral supplementation with *Lactobacillus* (LactoFem^®^, Takgene Zist Pharmaceutical Co., Tehran, Iran) significantly reduced symptoms of dysmenorrhea and chronic pelvic pain without concurrent hormonal treatment [182]. These results indicate that Lactobacillus probiotics may provide a non-hormonal, low-risk supplementary treatment for managing pain in endometriosis. Research involving animals further substantiates these findings, demonstrating that providing Lactobacillus orally to mice can diminish the size of endometriotic lesions by boosting the function of NK cells and elevating cytokine production such as IL-6 and IL-12 [183,184,185,186]. Given that diminished NK cell function is a characteristic of immune dysregulation linked to endometriosis, probiotics might help regain immune monitoring and lower lesion growth.

Significantly, the preventive capability of *Lactobacillus* in halting the development of endometriotic lesions in animal studies indicates its importance in preventing recurrences. Together, these studies emphasize the combined diagnostic and therapeutic significance of gut microbiota in endometriosis and offer a basis for additional clinical trials focused on affirming microbiome-based treatments.

## 8. Clinical Trials on Gut Microbiota and EM

Research from various human studies has progressively underscored the unique changes in the microbiota within the gut, peritoneal fluid, and female reproductive system of those with endometriosis as opposed to healthy controls (Table 6) [187,188,189,190,191,192,193,194,195]. Despite these persuasive findings, it is still uncertain if these microbial changes are a contributing factor to disease development or just a reflection of the underlying condition [196,197,198,199]. Nonetheless, the repeated detection of microbiota imbalances in impacted individuals highlights its possible importance in the pathophysiology of endometriosis [200,201].

Hernandes et al. [191] performed a comparative study on microbial populations in vaginal fluid, eutopic endometrium, and endometriotic lesions from individuals with endometriosis, compared to those in healthy females. The research employed amplicon sequencing and found a broadly similar microbial profile among sample locations, with dominant genera such as *Lactobacillus*, *Gardnerella*, *Streptococcus*, and *Prevotella*. Nonetheless, a unique microbiota profile was discovered in deep endometriotic lesions, showing a decrease in *Lactobacillus* while *Alishewanella, Enterococcus*, and *Pseudomonas* were found in greater quantities. This implies that changes in microbes could be specific to certain lesions and localized in advanced disease scenarios.

In a separate study, Khan et al. [189] examined the treatment effects of antibiotics, with or without GnRHa, in females identified with endometriosis. The study showed a notable decrease in several pathogenic bacterial genera—such as *Gardnerella*, *Prevotella*, *Acidibacter*, *Atopobium*, *Megasphaera*, and *Bradyrhizobium*—by examining endometrial and endometriotic tissue samples from women treated with levofloxacin alone or alongside GnRHa. These microbial changes correlated with histological enhancements like reduced macrophage infiltration, lower cell proliferation, and diminished microvascular density, suggesting a positive impact on the inflammatory and angiogenic characteristics of the lesions.

A research project carried out by Wei et al. [190] concentrated on examining the spatial distribution of bacterial communities within the female reproductive tract. By analyzing samples from the lower reproductive tract (vagina and cervix) and the upper reproductive tract (endometrium and peritoneal fluid), the researchers noted a significant decrease in *Lactobacillus* and an increase in *Sphingobium* and *Pseudomonas viridiflava* in the upper areas of EM patients. These results indicate a potential upward pathway of microbial colonization linked to the advancement of endometriosis and provide a microbial signature that could aid in early identification or surveillance.

Chen et al. [192] aimed to create a microbiota profile that could differentiate between individuals with endometriosis, adenomyosis, and those with both disorders. Next-generation sequencing was employed to analyze cervical and posterior fornix swabs. The research revealed an increased occurrence of *Atopobium* in patients with both endometriosis and adenomyosis diagnoses. This microbial enrichment could indicate disease-specific inflammatory environments or hormonal influences, highlighting the potential of microbial screening to distinguish among complex gynecologic conditions.

In their study, Ata et al. [193] analyzed stool, vaginal, and endocervical samples to assess microbial communities in women with stage 3–4 endometriosis compared to healthy controls. While no significant differences were observed at the species level, minor distinctions at the genus level appeared. This indicates that although the total microbial diversity might stay consistent, specific taxa could show changes in abundance under particular conditions, warranting more detailed taxonomic and functional investigations in upcoming research. Akiyama et al. [194] studied cervical mucus samples from women both with and without endometriosis, revealing that *Streptococcus* and *Enterobacteriaceae* were more common in the EM group. These results bolster the theory that a localized microbial imbalance might affect mucosal immunity or lead to persistent inflammation, particularly in the cervical area. It also indicates a possible connection between the cervical microbiota and systemic effects of EM.

**Table 6:** Human clinical trials on gut microbiota and EM.

Study	Goals	Sample Size	Age Range, Years	Diagnosis Type	Sample Type	Techniques	Key Findings
Xiao et al. [187]	To analyze microbial diversity and relation with pain symptoms	40 cases/40 controls	19–43	Surgical confirmation	Fecal and cervical samples	16S rRNA sequencing	Distinct microbial signatures associated with pelvic pain severity
Lee et al., [188]	To evaluate correlation between gut dysbiosis and EM severity	35 cases/30 controls	20–42	Surgical confirmation	Fecal samples	Metagenomic sequencing	Reduced beneficial microbes and enriched pro-inflammatory taxa in EM
Khan et al., [189]	To evaluate effects of antibiotics ± GnRHa on EM microbiota and inflammation	53 cases/47 controls	18–51	Surgery and histology	Endometrial samples	NGS + Immunohistochemistry	Antibiotics reduced inflammation, pathogen load, and angiogenesis markers
Wei et al., [190]	To trace bacterial distribution in reproductive tract	36 cases/14 controls	23–44	Surgery and histology	Lower and upper reproductive tract	Ion Torrent PGM: V4-V5 16S rRNA	Lactobacillus decreased upward; Sphingobium, Pseudomonas found in uterus
Hernandes et al., 2020 [191]	To compare microbial profiles in vaginal and endometrial compartments	10 cases/11 controls	18–50	Surgery and histology	Vaginal fluid, endometrium, lesion tissue	NGS Illumina: V3-V4 rRNA	Deep lesions had more Alishewanella, Enterococcus, Pseudomonas
Chen et al., [192]	To identify microbiota associated with EM and adenomyosis	46 cases/36 controls	18–45	Surgery, ultrasound, MRI	Cervical and fornix swabs	NGS Illumina: V3-V4 16S rRNA	Atopobium enriched in EM–adenomyosis group
Ata et al., [193]	To compare gut, vaginal, and cervical microbiota between EM patients and controls	14 cases/14 controls	18–45	Surgery and histology (stage 3–4)	Stool, vaginal, endocervical swabs	NGS Illumina: V3-V4 16S rRNA	Minimal species-level differences; some genus-level shifts observed
Akiyama et al. [194]	To study microbiota patterns in cervical mucus of EM patients	30 cases/39 controls	20–44	Surgery and histology	Cervical mucus	NGS Illumina: V5-V6 16S rRNA	Streptococcus and Enterobacteriaceae more common in EM cervical mucus
Khan et al. [195]	To assess microbial colonization in intrauterine environment and ovarian cysts	32 cases/32 controls	21–52	Surgery and histology	Endometrial swabs, cystic fluid	NGS Illumina: 16S rRNA	Higher abundance of pathogens in cystic fluid; GnRHa lowered Lactobacillaceae

Collectively, these studies emphasize the increasing recognition that microbial populations in the reproductive system and intestines are closely connected to EM pathophysiology. They also emphasize the growing promise of microbiota-based diagnostics and supplementary therapies in addressing this intricate condition.

## 9. Limitations and Future Directions

Investigating the microbiota’s influence on EM is an emerging area, but various constraints impede the formation of conclusive findings. A main issue is the limited sample sizes common in numerous studies, which weaken the statistical power and generalizability of the results. For example, a narrative review pointed out that many studies on the gut and vaginal microbiota in EM had small sample sizes, limiting the strength of the findings. Future studies would gain from larger-scale multicenter research to improve the trustworthiness and relevance of the findings [202,203,204,205,206].

The diversity in the approaches to examining the microbial composition makes it more challenging to integrate data from different studies. Differences in DNA extraction methods, sequencing technologies, and bioinformatic evaluations can result in varied outcomes, complicating the task of pinpointing a reliable microbial signature linked to EM. Research comparing various sequencing techniques revealed considerable differences in microbial profiles, highlighting the necessity for standard protocols in microbiome studies. Creating standardized methods would enable more precise comparisons across studies and enhance meta-analyses [207,208,209,210,211,212,213,214,215].

The intrusive characteristics of existing diagnostic methods for EM, like laparoscopy, create ethical and practical difficulties in enlisting asymptomatic healthy participants for comparative research. This restriction biases the accessible data towards symptomatic groups, possibly neglecting the wider range of microbiota variations. Creating non-invasive diagnostic methods, such as microbiome-based tests, may alleviate this problem. Recent studies indicate that certain microbial indicators in the reproductive system could act as possible non-invasive biomarkers for EM. The additional confirmation of these biomarkers could transform diagnostic methods and enable wider participant inclusion in the research [216,217,218,219].

The nature of the relationship between microbiota dysbiosis and EM is still uncertain. It remains unclear whether changes in the microbiome play a role in the pathogenesis of EM or are a consequence of the disease. Longitudinal studies are crucial for clarifying causal relationships and time-related changes. For instance, studies have shown that the gut microbiota can affect estrogen metabolism, potentially contributing to the development of EM. Grasping these interactions throughout time may guide focused interventions [220,221].

## 10. Conclusions

EM is a multifaceted, estrogen-dependent inflammatory condition that impacts millions of women globally; however, its exact pathogenesis remains inadequately elucidated. Emerging research indicates a substantial involvement of gut microbiota in the initiation and advancement of EM through mechanisms of immune modulation, metabolic changes, and the metabolism of estrogen. Investigations have revealed that the dysbiosis of the gut microbiota can affect systemic inflammatory responses, enhance oxidative stress, and disrupt immune homeostasis, thus intensifying the proliferation of endometriotic lesions. Furthermore, metabolites derived from microbiota, LPS and SCFAs, play a role in the pathophysiology of the disease by either amplifying inflammatory signaling pathways or providing protective anti-inflammatory effects.

The association between the gut microbiota and EM is further substantiated by studies that have identified microbial biomarkers with prospective diagnostic significance. Changes in the microbial diversity, such as diminished *Lachnospiraceae Ruminococcus* and heightened levels of *Escherichia/Shigella*, have been correlated with the severity of the disease, indicating their potential utility as non-invasive biomarkers for the diagnosis of EM. Additionally, the gut microbiota are instrumental in the metabolism of estrogen through the estrobolome, which affects estrogen circulation and may contribute to the progression of EM. These findings suggest that gut microbiota could be a pivotal target for innovative therapeutic strategies.

Beyond diagnostic applications, therapeutic strategies aimed at modulating gut microbiota show potential for the management of EM. Probiotics, particularly *Lactobacillus gasseri* OLL2809, have demonstrated an ability to inhibit lesion growth and alleviate pain associated with EM. Dietary interventions, such as the supplementation of PUFAs and adherence to low-FODMAP diets, have shown potential in modifying the composition of the gut microbiota and attenuating inflammatory responses. Furthermore, FMT emerges as a novel and promising strategy that warrants further exploration as a potential therapeutic option for EM.

Despite these advancements, significant obstacles persist. The heterogeneity in the gut microbiota composition, attributable to genetic, environmental, and lifestyle variables, complicates the establishment of a definitive microbial signature indicative of EM. Moreover, a majority of existing studies depend on limited sample sizes, animal models, or observational data, emphasizing the need for large-scale, multi-center clinical trials to corroborate these findings. Additionally, advancements in bioinformatics, including deep learning predictive algorithms and single-cell RNA sequencing, may enhance our capacity to identify critical microbial markers and develop precision medicine strategies tailored to individual patients.

In conclusion, the complex interactions between the gut microbiota, immune regulation, and estrogen metabolism underscore their essential role in the pathophysiology of EM. Continued research that integrates microbiome analysis, metabolomics, and bioinformatics is crucial for the advancement of microbiota-based diagnostic modalities and therapeutic interventions. Future endeavors should concentrate on refining microbiome-targeted therapies to yield safer, more efficacious, and personalized solutions for women afflicted with EM.

## Figures and Tables

**Table 3:** Gut microbiota aspects with EM [87,88,89,90,91,92,93,94,95].

Aspect	Key Findings
Role of gut microbiota in EM	Gut microbiota influence immune responses, estrogen metabolism, and inflammation, playing a crucial role in EM progression.
Microbial dysbiosis in EM	Studies have shown altered microbial composition in EM patients, with decreased *Ruminococcaceae* and increased *Streptococcus*, *Blautia*, *Dorea*, and *E. coli.*
Gut barrier disruption	Gut dysbiosis leads to compromised gut barrier integrity, allowing bacterial metabolites (LPS) to enter circulation and trigger immune activation.
Immune system activation	Increased endotoxin (LPS) levels activate TLR4, promoting the secretion of pro-inflammatory cytokines such as IL-6, TNF-*α*, and IL-8.
Inflammatory pathways	EM patients exhibit overexpression of NF-κB, IL-1, IL-8, and COX-2, leading to chronic pelvic inflammation and pain.
Impact of antibiotics	Broad-spectrum antibiotics and metronidazole have been shown to reduce lesion size and inflammatory markers in EM mouse models.
FMT	FMT from EM-induced mice restored lesion growth and inflammation in antibiotic-treated mice, highlighting the direct role of gut microbiota in EM.
Estrogen–microbiota interaction	Gut microbiota modulate estrogen metabolism by regulating *β*-glucuronidase activity, which influences estrogen reabsorption and systemic estrogen levels.
Protective microbes	*Lactobacillus* and *Bifidobacterium* help reduce *β*-glucuronidase-producing bacteria, potentially mitigating EM progression.
Potential therapeutic approaches	Probiotics, prebiotics, FMT, and COX-2 inhibitors have been suggested as possible interventions to target microbiota-mediated EM progression.

**Table 4:** Therapeutic windows and surgical management of EM [149,153,154,155,156].

Aspect	Findings	Evidence Quality	Limitations	Clinical Implications	Future Research Needs	Recommended Actions
Window of opportunity	No clearly defined period for optimal intervention.	No strong evidence	Lack of preventive treatments and monitoring tools.	Difficulty in early intervention and disease management.	Development of early detection biomarkers.	Prioritize biomarker discovery in clinical setting
Recurrence management	No established treatment for recurrence post-surgery.	Weak	No consensus on defining recurrence.	Need for individualized follow-up strategies.	Improved non-invasive diagnostic techniques.	Use symptom tracking tools for individualized care
Surgical treatment	Laparoscopic removal improves pain relief in 6 months.	Moderate (RCTs, *n* = 171)	Limited number of studies; mixed EM subtypes.	Surgery still recommended for symptom relief.	Larger RCTs comparing surgical techniques.	Standardize surgical techniques and outcome metrics
Long-term surgical outcomes	One RCT suggests benefits up to 12 months post-surgery.	Low	Small sample size (*n* = 69), limited follow-up data.	Uncertainty about long-term benefits.	Need for long-term follow-up studies.	Monitor long-term outcomes post-surgery systematically
Superficial peritoneal EM	Limited evidence that surgery improves symptoms.	Uncertain	Lack of RCTs comparing surgery vs. non-surgical treatment.	Consideration of alternative medical management.	Comparison of ablation vs. excision effectiveness.	Evaluate non-surgical therapies in comparative studies
Ovarian EM	Surgical excision preferred, especially for large cysts.	Moderate	No threshold cyst size for surgery identified.	Tissue analysis necessary to rule out malignancy.	Need for ovarian reserve preservation strategies.	Develop clear surgical criteria and ovarian function assessment
Impact on fertility	Surgery may damage ovarian reserve, affecting fertility.	Insufficient data	Unknown impact of cyst excision on ovarian function.	Risk assessment needed before surgical intervention.	Fertility preservation methods in EM patients.	Implement fertility counseling and AMH monitoring protocols
Deep EM treatment	Surgery is the primary treatment option.	Strong	Some patients benefit from medical therapy instead.	Individualized decision making based on symptom severity.	Optimization of medical vs. surgical treatment balance.	Use shared decision-making models for treatment choice

**Table 5:** Advances and challenges in gut microbiota research related to EM [147,149,153].

Research Focus	Key Insights	Limitations
Correlation between gut microbiota and EM	Ongoing studies explore the relationship between gut microbiota composition and EM pathogenesis.	Further systematic research is needed to establish causality and precise mechanisms.
Advancements in genome sequencing	Modern sequencing technologies have significantly advanced microbiota research, improving understanding within a short period.	The complexity and vast number of gut microorganisms make it challenging to detect all species, leading to potential gaps in data.
Microbiome-derived metabolites	Investigations suggest that microbiota-derived metabolites may play a key role in EM progression and treatment potential.	The specific metabolic pathways through which microbiota influence EM remain unclear, requiring more research.
Histological subtypes of EM	EM consists of three main types: superficial peritoneal EM, deeply infiltrating EM, and ovarian EM, each with varying clinical manifestations.	EM lesions coexist with heterogeneous symptoms, making diagnosis and treatment complex.
Microbiota-mediated pain and infertility	Future research should focus on how specific microbiota or metabolites contribute to pelvic pain and infertility in EM patients.	A lack of precise biomarkers complicates the differentiation between pain-dominant and infertility-dominant EM cases.
Early and sensitive diagnostic methods	Identifying microbial markers could lead to earlier, safer, and more effective non-invasive diagnostic techniques.	The diagnostic value of gut microbiota remains unclear and requires validation in large-scale clinical studies.
Bioinformatics and predictive models	Single-cell RNA sequencing, deep learning models, and continuously updated microbiome databases are promising tools for risk stratification and early screening.	Integrating microbiota data with clinical diagnostics remains a challenge, and predictive accuracy requires further refinement.
Microbiota-based therapies	Targeting specific gut microorganisms may lead to novel interventions with fewer side effects than existing treatments.	Current medical and surgical treatments for EM have high recurrence rates; microbial interventions need extensive clinical validation before widespread use.
Drug metabolism and microbiome interactions	Understanding how gut microbiota influence drug metabolism could optimize personalized treatment strategies.	Drug–microbiome interactions are highly individualized, requiring personalized medicine approaches.

## Abbreviations

The following abbreviations are used in this manuscript:

EM	Endometriosis
WHO	World Health Organization
EDCs	Endocrine-Disrupting Chemicals
PCOS	Polycystic Ovary Syndrome
IBD	Inflammatory Bowel Disease
MD	Microbiota-Depleted
PubMed	Public/Publisher Medline
WoS	Web Of Science
JSTOR	Journal Storage
BASE	Bielefeld Academic Search Engine
NAFLD	Non-Alcoholic Fatty Liver Disease
IBD	Inflammatory Bowel Disease
CVD	Cardiovascular Disease
ALD	Alcoholic Liver Disease
CKD	Chronic Kidney Disease
PID	Pelvic Inflammatory Disease
RNA	Ribonucleic Acid
16S rRNA	16s Ribosomal Ribonucleic Acid
FDR	False Discovery Rate
PCR	Polymerase Chain Reaction
IL	Interleukin
SCFAs	Short-Chain Fatty Acids
RIG-I	Retinoic Acid-Inducible Gene I
NF-kB	Nuclear Factor Kappa-Light-Chain-Enhancer Of Activated B Cells
TNF-α	Tumor Necrosis Factor-Alpha
Bax	Bcl-2-Associated X Protein
Fas	Fas Cell Surface Death Receptor
VEGF	Vascular Endothelial Growth Factor
CDCA	Chenodeoxycholic Acid
UDCA	Ursodeoxycholic Acid
GPCRs	G-Protein-Coupled Receptors
HDACs	Histone Deacetylases
GALT	Gastrointestinal-Associated Lymphoid Tissue
Tregs	Regulatory T Cells
PAMPs	Pathogen-Associated Molecular Patterns
LPS	Lipopolysaccharides
PRRs	Pattern Recognition Receptors
TLRs	Toll-Like Receptors
IECs	Intestinal Epithelial Cells
SCF	Stem Cell Factor
G-CSF	Granulocyte Colony-Stimulating Factor
SFB	Segmented Filamentous Bacteria
TLR4	Toll-Like Receptor 4
NLRP3	NOD-like Receptor Pyrin Domain Containing 3
FMT	Fecal Microbiota Transplantation
COX-2	Cyclooxygenase-2
PGE2	Prostaglandin E2
ER*α*	Estrogen Receptor Alpha
ER*β*	Estrogen Receptor Beta
GPER1	G Protein-Coupled Estrogen Receptor
EREs	Estrogen Response Elements
AP-1	Activator Protein-1
SP-1	Stimulating Protein-1
MAPK	Mitogen-Activated Protein Kinase
PI3K	Phosphoinositide 3-Kinase
cAMP	Cyclic Adenosine Monophosphate
EAOC	EM-Associated Ovarian Cancer
CCC	Clear Cell Carcinoma
OEC	Ovarian Endometrioid Carcinoma
AKT	Protein Kinase B (also known as AKT)
mTOR	Mechanistic Target of Rapamycin
MR	Mendelian randomization
NSAIDs	Nonsteroidal Anti-Inflammatory Drugs
GnRH	Gonadotropin-Releasing Hormone
NK	Natural Killer
PUFAs	Polyunsaturated Fatty Acids
DHA	Docosahexaenoic Acid
DPA	Docosapentaenoic Acid
EPA	Eicosapentaenoic Acid
*E. coli*	*Escherichia coli*
FODMAP diet	Fermentable Oligo-, Di-, Monosaccharides, And Polyols diet
CDI	*Clostridium difficile* infections
RCTs	Randomized Controlled Trials
